# Changes in cardiac functions in patients treated with parathyroidectomy for secondary hyperparathyroidism

**DOI:** 10.1007/s13304-024-01812-8

**Published:** 2024-03-26

**Authors:** Sami Benli, Emrah Yesil, Deniz Tazeoglu, Cumhur Ozcan, Ismail Turkay Ozcan, Ahmet Dag

**Affiliations:** 1 Department of General Surgery, Division of Surgical Oncology, Mersin University Medical Faculty, Mersin, Turkey; 2 Department of Cardiology, Mersin University Medical Faculty, Mersin, Turkey; 3 Department of General Surgery, Division of Endocrine Surgery, Mersin University Medical Faculty, Mersin, Turkey

**Keywords:** Secondary hyperparathyroidism, Myocardial performance index, Tei index, Cardiac function

## Abstract

Our study aims to investigate the changes in cardiac functions, especially myocardial performance index (MPI), in patients who underwent parathyroidectomy for secondary hyperparathyroidism. Patients who underwent parathyroidectomy for secondary hyperparathyroidism between June 2010 and September 2021 were analyzed retrospectively. The patients were divided into two groups: those who underwent total parathyroidectomy (group 1) and those who underwent subtotal parathyroidectomy (group 2). The groups were compared according to the echocardiogram findings performed in the preoperative period and the postoperative sixth month. In addition, cardiac structure, and systolic and diastolic functions, especially myocardial performance index, were evaluated by echocardiography and Doppler imaging. Thirty-seven patients were examined; 16 (43.2%) underwent total parathyroidectomy, and 21 (56.8%) had subtotal parathyroidectomy performed. Group 1’s mean left ventricular end-systolic diameter (LVES) decreased from 2.53 ± 0.57 to 2.35 ± 0.37 cm after parathyroidectomy. In Group 1, the postoperative value of LVES and end-systolic volume decreased significantly compared to the preoperative period (*p* = 0.042, *p* = 0.008, respectively). EF increased from 59.25 ± 0.05 to 67.81 ± 4.04. In Group 1, EF and EV postoperatively increased significantly compared to the preoperative period (*p* = 0.023, *p* = 0.021, respectively). The mean MPI decreased from 0.45 ± 0.07 to 0.39 ± 0.04 after parathyroidectomy in group 1. In group 2, it decreased from 0.46 ± 0.06 to 0.40 ± 0.04 (*p* < 0.001). The present study provides an improvement in myocardial functions after parathyroidectomy. While LVES, EF, ejection volume, end-systolic volume, and MPI improved in both groups, the MPI improvement was more evident in the total parathyroidectomy group.

**Graphical Abstract:**

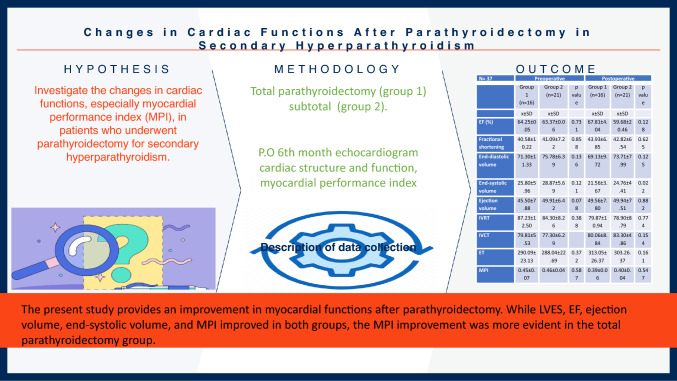

## Introduction

Secondary hyperparathyroidism (SHPT) is a process aimed at providing vitamin and mineral homeostasis by increasing the proliferation of parathyroid cells and hormone secretion by decreased calcium and vitamin D synthesis due to phosphorus uptake in advanced stages of chronic kidney disease (CKD) [1]. In addition to CKD, SHPT is also seen in malabsorption, vitamin D deficiency, and pseudohypoparathyroidism [2].

It is known that elevated parathyroid hormone (PTH) levels are associated with various cardiovascular diseases (CVD). Prolonged SHPT causes visceral and vascular calcifications due to disorders in the bone mineral cycle, leading to cardiovascular morbidity and mortality [3]. According to the data obtained based on the U.S Renal Data System registry data, it has been shown that an intact parathyroid hormone (IPTH) level of 91–197 pg/mL versus > 495 pg/mL is associated with a higher risk of sudden death than CVDs [4]. Therefore, keeping calcium and phosphorus ratios at certain values in SHPT is very important to minimize cardiovascular risks. Although there are medical treatment alternatives, such as phosphate binders, calcimimetics, and vitamin D sterols/analogs in mild and moderate SHPT, the target PTH value can be kept below <300 pg/ml in only 22% of patients in severe SHPT [5]. For these reasons, surgical parathyroidectomy (PTx) is the most crucial step in treating severe SHPT patients. The type of surgical technique to be performed in secondary hyperparathyroidism depends on the clinicopathological characteristics of the patients and the experience of the surgeon; it can be total PTx, total PTx with autotransplantation, and subtotal PTx [6].

Especially in CKD patients, good results are seen in biochemical parameters, cardiovascular functions, and mortality [7, 8] related to bone and mineral metabolism after PTx. Furthermore, positive effects on the cardiac system have been shown due to decreased cardiovascular calcifications, improved blood lipid profile, normalization of blood pressure, and improved anemia after PTx [9]. In addition, cardiac hypertrophy is observed after PTx [10]; in this case, it affects the recovery of cardiac functions. The reason for cardiac hypertrophy is the decrease in Fibroblast Growth Factor-23 level after PTx [11].

MPI is a measurement method that evaluates the systolic and diastolic functions of the ventricles together without being affected by blood pressure, age, heart rate, and ventricular geometry [12]. It has been shown that elevation in MPI is an independent indicator of cardiac death in heart diseases [13]. Although the effect of parathyroidectomy on left ventricle functions in SHPT is not fully understood, it has been reported that there is a 37–41% reduction in cardiovascular mortality of patients after effective PTX [14]. Therefore, the present study aims to evaluate the changes in cardiac functions, especially myocardial performance index, in the pre- and post-PTx period in SHPT patients in line with echocardiography findings.

## Material and method

### Patient selection

The Mersin University Ethics Committee approved this study protocol under the number E-78017789-050.01.04-1638902. The study has been registered on the ClinicalTrial.gov website under the identification number NCT06187480.

The patients who developed secondary hyperparathyroidism after chronic renal failure and underwent subtotal/total parathyroidectomy in our hospital between June 2010 and September 2021 were analyzed with a single-center Nested case–control study design.

Patients with a diagnosis of chronic renal failure, secondary hyperthyroidism after chronic renal failure, preoperative and postoperative laboratory tests, parathyroidectomy operation, and preoperative and postoperative 6-month transthoracic echocardiography (ECHO) were included in the study.

Advanced-stage lung disease, atrial fibrillation, atrial and supraventricular tachycardia, extra-ventricular beats, intraventricular conduction disorders, ventricular pacing, moderate to severe valve pathology, history of bypass surgery, poor image quality in transthoracic echocardiography as cardiac comorbidities, patients with disease and missing data were excluded from the study.

### Data collection

Demographic data (age, gender), surgery (total/subtotal parathyroidectomy), preoperative and postoperative laboratory analyses, and ECHO reports of the patients were recorded. In laboratory values, glucose, hemoglobin, albumin, blood urea nitrogen (BUN), creatinine, calcium, phosphorus, and parathormone values were recorded. In ECHO reports, left ventricular end-diastolic diameter (cm), left ventricular end-systolic diameter (cm), left ventricular end-diastolic septum thickness (cm), left ventricular end-diastolic posterior wall thickness (cm), ejection fraction (%), fractional shortening, end-diastolic volume, end-systolic volume, ejection volume, isovolumetric relaxation time (IVRT), isovolumetric contraction time (IVCT), ejection time (ET), and myocardial performance index (MPI) data were recorded.

### Surgical technique

Patients resistant to calcimimetics and vitamin D analogs, patients with symptoms, such as bone and joint pain, persistent itching, and muscle aches, and patients whose symptoms affect their quality of life are among the indications for surgery. In addition, parathyroidectomy was performed on patients with uncontrolled hyperphosphatemia, anemia hyporesponsive to erythropoietin therapy, hypercalcemia, and vascular and tissue calcifications, considered complications of SHPT.

Subtotal or total parathyroidectomy is performed in the SHPT. Total parathyroidectomy was performed to prevent recurrent resistant SHPT in patients with long life expectancy and little or no possibility of kidney transplantation. In the preoperative period, routine ultrasound and 99mTc-sestamibi scintigraphy were performed on the patients. During neck exploration, tissues considered the parathyroid gland were examined as a frozen section. As a result of the frozen section, the number of glands removed was confirmed. After parathyroidectomy was completed, intraoperative parathormone testing was performed in all patients, and it was observed that parathormone levels decreased. In the subtotal parathyroidectomy procedure, four parathyroid glands are explored. Usually, half of the parathyroid gland, closest to normal in appearance and size, is left in place with its vascular network. Other glands are excised. Four parathyroid glands are explored and excised in the total parathyroidectomy with autotransplanted procedure. Then, a part of the parathyroid gland closest to normal in appearance and size is auto-transplanted into the sternocleidomastoid muscle or the forearm. The transplantation area is marked with a metal clip.

### Echocardiography (ECHO)

All patients were evaluated with two-dimensional, pulse-wave Doppler, and tissue Doppler echocardiography. Philips HD11 XE device was used to detect echocardiographic data. Parasternal long axis view with M-Mode echocardiography method left ventricular interventricular septum thickness, left ventricular posterior wall thickness, left ventricular end-diastolic diameter, left ventricular end-systolic diameter, left ventricular end-diastolic volume, left ventricular end-systolic volume, left ventricular end-systolic volume, and fractional shortening data was determined. In addition, left ventricular stroke volume was determined by subtracting the left ventricular end-systolic volume from the left ventricular end-diastolic volume.

### Myocardial performance index calculation technique

The myocardial performance index (MPI) is a numerical value obtained using cardiac time intervals. This numerical value was obtained by dividing the sum of isovolumetric contraction time (ICT) and isovolumetric relaxation time (IRT) by ejection time (ET). MPI can be detected using conventional Pulse-wave Doppler or tissue Doppler echocardiography. The mean average left ventricular (LV) MPI value is 0.39 ± 0.05. In adults, LV MPI values less than 0.40 are considered normal. Higher index values are associated with cardiac dysfunction [15].

**Fig. 1:**
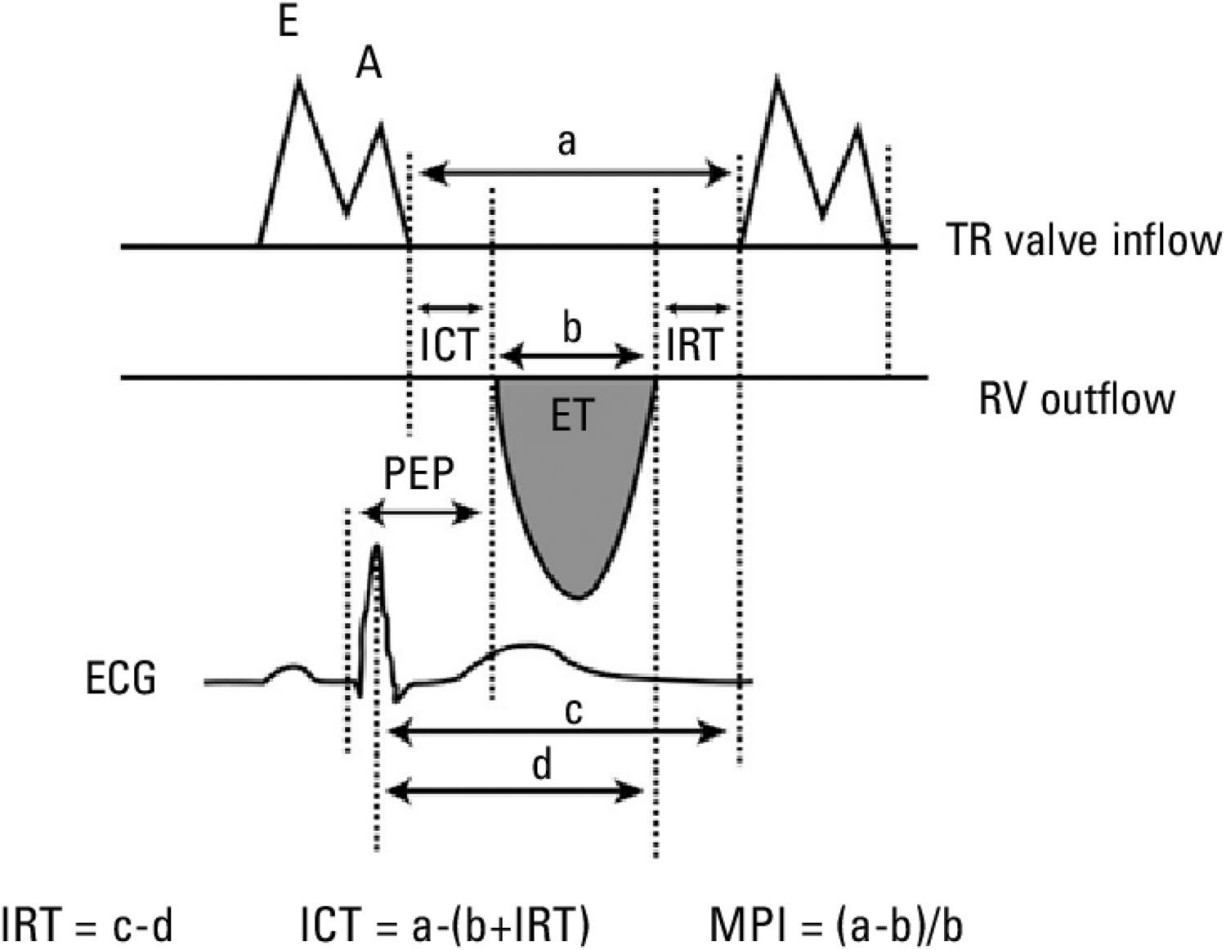
Myocardial performance index calculation. Measurement of right ventricular pre-ejection period (PEP), ejection time (ET), isovolumetric contraction time (ICT), isovolumetric relaxation time (IRT), and calculation of myocardial performance index (MPI)

Our study obtained cardiac time intervals using Pulsed wave Doppler echocardiography techniques. MPI, Pulsed wave Doppler echocardiography from the apical four-chamber images, by placing the sample volume at the endpoints of the mitral valves, the time interval between the end and the beginning of the mitral flow (a), switching to the apical five-chamber images and placing the sample volume in the left ventricular outflow tract just below the aortic valves to determine the left ventricular ET (b) was done. The left ventricular isovolumetric (ICT + IRT) sum was calculated by subtracting left ventricular ET from the time interval between the end and beginning of mitral flow (a-b). Thus, MPI [(a-b)/b] was obtained [16] (Fig. 1).

### Statistical analysis

Mean and standard deviation were used for the statistics of continuous data. Median, minimum, and maximum values were used for continuous or ordinal data that did not show normal distribution. Frequency (*n*) and percentage (%) values were used to define categorical variables. Fisher’s exact test was used to compare the means of two independent groups. The chi-square test was used to evaluate the relationship between categorical variables. A paired t-test was used to compare the patients’ preoperative and postoperative numerical data. The statistical significance level of the data was taken as *p* < 0.05. The statistical program www.e-picos.com was used to evaluate the data.

## Results

**Fig. 2:**
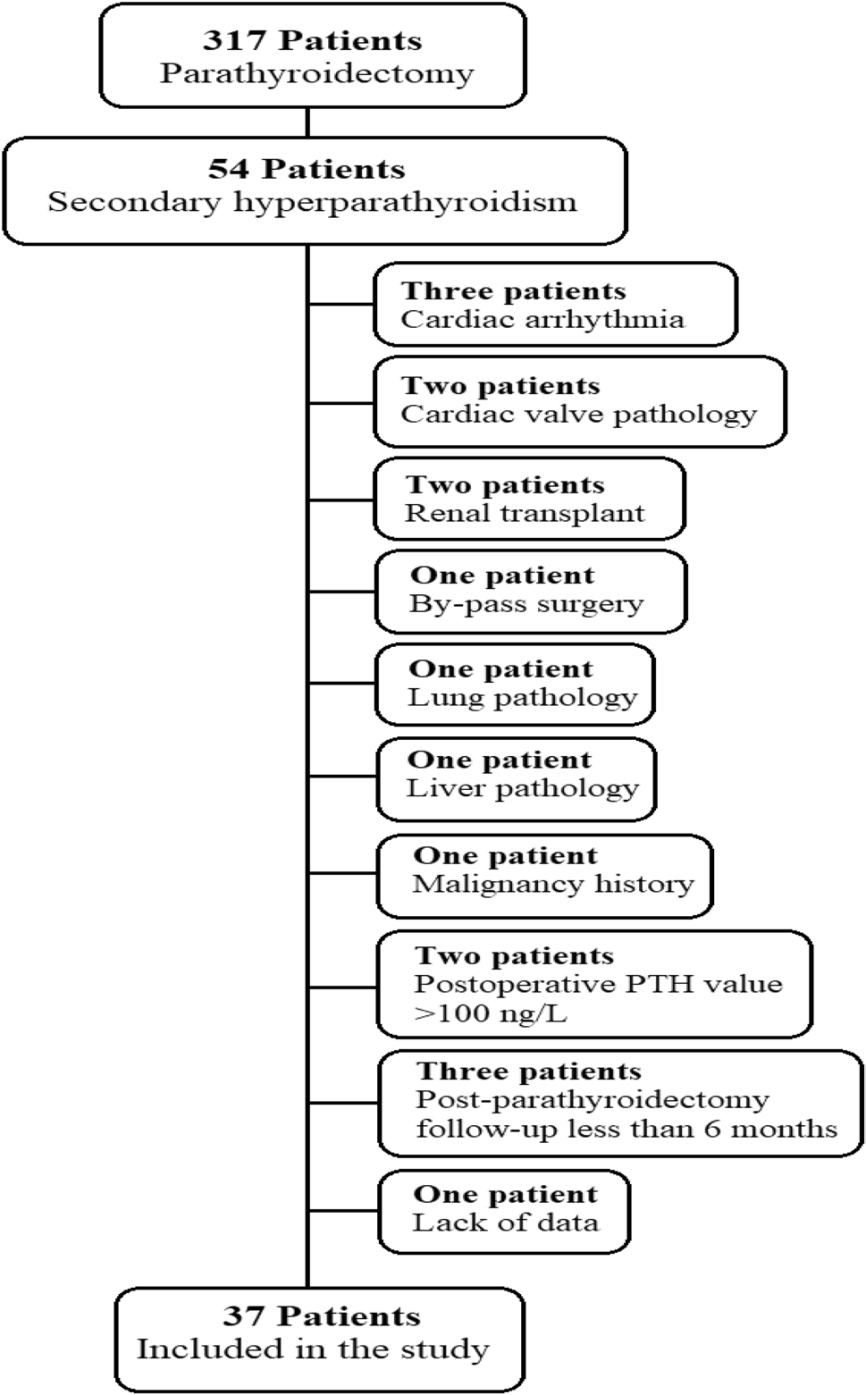
Flow chart of the study

During the study, parathyroidectomy was performed on 317 patients in our hospital. Parathyroidectomy was performed in 54 (17%) patients diagnosed with secondary hyperparathyroidism. Fifty-four patients who underwent subtotal or total parathyroidectomy were analyzed. Three patients had cardiac arrhythmia, two patients had heart valve pathology, two patients had renal transplantation, one patient had a history of bypass surgery, one patient had lung pathology, one patient had liver pathology, one patient had a history of malignancy, two patients had a PTH value >100 ng/L after parathyroidectomy, and three patients follow-up period after parathyroidectomy was shorter than 6 months and one patient was excluded because of missing data. Thirty-seven patients were included in the study (Fig. 2).

The mean age of the patients was 46.75 ± 13.19 years. Ten (27%) patients were female, and 27 (73%) were male. Total parathyroidectomy was performed in 16 (43.2%) patients, and subtotal parathyroidectomy was performed in 21 (56.8%) patients. The patients were grouped and compared according to the surgical technique (Table 1).

**Table 1:** Demographic and clinical data of the patients

	Group 1 16 (43.2%)	Group 2 21 (56.8%)	All patient (*n* = 37)
			*x* ± SD
Age (mean. SD) Median (min–max)	45.89 ± 12.56	46.83 ± 12.21	46.70 ± 13.19 44 (20–72)

			*n* (%)
*Gender*			
Female	4	6	10 (27)
Male	10	17	27 (73)
*Preoperative biochemistry*			
Glucose (mg/dL)	93.50 ± 12.57	96.05 ± 27.69	94.77 ± 20.13
Hgb (g/dL)	10.8 ± 1.9	11.1 ± 1.7	11.0 ± 1.8
Albumin (g/dL)	3.62 ± 0.24	3.55 ± 0.69	3.58 ± 0.46
BUN (mg/dL)	96.69 ± 19.46	98.88 ± 33.35	98.88 ± 33.35
Cr (mg/dL)	8.11 ± 2.42	8.41 ± 3.01	8.26 ± 2.71
Ca (mg/dL)	9.58 ± 0.81	9.11 ± 1.49	9.34 ± 1.0
P (mg/dL)	5.55 ± 1.14	5.74 ± 2.19	5.65 ± 1.7
PTH (ng/L)	1443.9 ± 695.1	1671.5 ± 714.0	1557.6 ± 704.5
*Echocardiography findings*			
LVED diameter (cm)	4.09 ± 0.22	4.26 ± 0.59	4.17 ± 0.41
LVES diameter (cm)	2.53 ± 0.57	2.62 ± 0.47	2.58 ± 0.52
LVED septum thickness (cm)	1.09 ± 0.14	1.11 ± 0.15	1.1 ± 0.15
LVED posterior wall thickness (cm)	1.05 ± 0.15	1.04 ± 0.21	1.05 ± 0.18
EF (%)	64.25 ± 0.05	63.37 ± 0.06	63.81 ± 0.06
Fractional shortening	40.58 ± 10.22	41.09 ± 7.22	40.83 ± 8.72
End-diastolic volume	71.30 ± 11.33	75.78 ± 6.39	73.54 ± 8.86
End-systolic volume	25.80 ± 5.96	28.87 ± 5.69	27.33 ± 5.82
Ejection volume	45.50 ± 7.88	49.91 ± 6.42	47.70 ± 7.15
IVRT	87.23 ± 12.50	84.30 ± 8.26	85.77 ± 10.38
IVCT	79.81 ± 5.53	77.30 ± 6.29	78.6 ± 5.91
ET	290.1 ± 23.13	288.0 ± 22.69	289.0 ± 22.9
MPI	0.45 ± 0.07	0.46 ± 0.04	0.46 ± 0.05

The mean age was 45.89 ± 12.56 years in group 1 (total parathyroidectomy) and 46.83 ± 12.21 years in group 2 (subtotal parathyroidectomy). There were 4 (25%) women in group 1 and 6 (28.6%) in group 2. Hemodialysis was applied to all patients 3 times a week. There was no statistically significant difference between the groups regarding age and gender distribution (*p* > 0.05).

The groups’ preoperative and postoperative blood tests and ECHO findings are shown in Table 2. The preoperative (laboratory results and ECHO findings) and postoperative data of the patients were compared according to the groups. There was a statistically significant decrease in Ca (*p* < 0.001), P (*p* < 0.001), and PTH (*p* < 0.001) values in the postoperative period compared to the preoperative period in both groups. The mean end-systolic volume in the postoperative period was 21.56 ± 3.67 in group 1 and 24.76 ± 4.41 in group 2. While there was no significant difference between the groups in terms of end-systolic volume mean in the preoperative period, it was significantly less in group 1 compared to group 2 in the postoperative period (*p* = 0.022). There was no statistically significant difference between the groups in terms of other laboratory results and ECHO findings in the preoperative and postoperative periods (*p* > 0.05) (Table 2).

**Table 2:** Comparison of preoperative and postoperative laboratory and echocardiographic results of the groups

*N* = 37	Preoperative			Postoperative		
	Group 1 (*n* = 16)	Group 2 (*n* = 21)	*p* value	Group 1 (*n* = 16)	Group 2 (*n* = 21)	*p* value
	*x* ± SD	*x* ± SD		*x* ± SD	*x* ± SD	
*Biochemistry*						
Glucose (mg/dL)	93.50 ± 12.57	96.05 ± 27.69	0.686	95.56 ± 24.54	96.28 ± 13.41	0.910
Hgb (g/dL)	10.83 ± 1.96	11.15 ± 1.75	0.604	11.03 ± 1.51	11.17 ± 2.28	0.833
Albumin (g/dL)	3.62 ± 0.24	3.55 ± 0.69	0.701	3.75 ± 0.33	3.64 ± 0.66	0.546
BUN (mg/dL)	96.69 ± 19.46	98.88 ± 33.35	0.817	90.74 ± 19.57	97.85 ± 34.81	0.751
Cr (mg/dL)	8.11 ± 2.42	8.41 ± 3.01	0.746	7.86 ± 1.98	8.04 ± 2.18	0.794
Ca (mg/dL)	9.58 ± 0.81	9.11 ± 1.49	0.263	7.64 ± 1.09	8.12 ± 0.88	0.147
P (mg/dL)	5.55 ± 1.14	5.74 ± 2.19	0.968	4.34 ± 0.89	4.72 ± 0.91	0.212
PTH (ng/L)	1443.87 ± 695.10	1671.48 ± 714.02	0.338	36.75 ± 18.74	54.48 ± 20.63	0.268
*Echocardiography findings*						
LVED diameter (cm)	4.09 ± 0.22	4.26 ± 0.59	0.282	4.42 ± 0.23	4.38 ± 0.70	0.828
LVES diameter (cm)	2.53 ± 0.57	2.62 ± 0.47	0.566	2.35 ± 0.37	2.42 ± 0.34	0.327
LVED septum thickness (cm)	1.09 ± 0.14	1.11 ± 0.15	0.654	1.11 ± 0.15	1.13 ± 0.13	0.734
LVED posterior wall thickness (cm)	1.05 ± 0.15	1.04 ± 0.21	0.496	1.00 ± 0.15	0.96 ± 0.36	0.671
EF (%)	64.25 ± 0.05	63.37 ± 0.06	0.731	67.81 ± 4.04	59.68 ± 20.46	0.128
Fractional shortening	40.58 ± 10.22	41.09 ± 7.22	0.858	43.93 ± 6.85	42.82 ± 6.54	0.625
End-diastolic volume	71.30 ± 11.33	75.78 ± 6.39	0.136	69.13 ± 9.72	73.71 ± 7.99	0.125
End-systolic volume	25.80 ± 5.96	28.87 ± 5.69	0.121	21.56 ± 3.67	24.76 ± 4.41	**0.022**
Ejection volume	45.50 ± 7.88	49.91 ± 6.42	0.078	49.56 ± 7.80	49.94 ± 7.51	0.882
IVRT	87.23 ± 12.50	84.30 ± 8.26	0.388	79.87 ± 10.94	78.90 ± 8.79	0.774
IVCT	79.81 ± 5.53	77.30 ± 6.29		80.06 ± 8.84	83.30 ± 4.86	0.154
ET	290.09 ± 23.13	288.04 ± 22.69	0.372	313.05 ± 26.37	303.26.37	0.161
MPI	0.45 ± 0.07	0.46 ± 0.04	0.587	0.39 ± 0.06	0.40 ± 0.04	0.547

*Hgb* hemoglobin, *BUN* blood urea nitrojen, *Cr* kreatinin, *Ca* calcium, *P* phosphorus, *PTH* parathormone, *LVES* left ventricular end-systolic, *LVED* left ventricular end-diastolic, *EF* ejection fraction, *IVRT* isovolumetric relaxation time, *IVCT* isovolumetric contraction time, *ET* ejection time, *MPI* myocardial performance index

In Group 1, the mean LVES was 2.53 ± 0.57 preoperatively and 2.35 ± 0.37 cm postoperatively. The mean end-systolic volume was 25.80 ± 21.56 preoperatively and 21.56 ± 3.67 postoperatively. In Group 1, the postoperative value of LVES and end-systolic volume decreased significantly compared to the preoperative period (*p* = 0.042, *p* = 0.008, respectively) (Table 3).

**Table 3:** Comparison of preoperative and postoperative results within the groups

*N* = 37	Group 1 (*n* = 16)			Group 2 (*n* = 21)		
	Preoperative	Postoperative	*p* value	Preoperative	Postoperative	*p* value
	*x* ± SD	*x* ± SD		*x* ± SD	*x* ± SD	
*Biochemistry*						
Glucose (mg/dL)	93.50 ± 12.57	95.56 ± 24.54	0.768	96.05 ± 27.69	96.28 ± 13.41	0.871
Hgb (g/dL)	10.83 ± 1.96	11.03 ± 1.51	0.587	11.15 ± 1.75	11.17 ± 2.28	0.912
Albumin (g/dL)	3.62 ± 0.24	3.75 ± 0.33	0.212	3.55 ± 0.69	3.64 ± 0.66	0.668
BUN (mg/dL)	96.69 ± 19.46	90.74 ± 19.57	0.394	98.88 ± 33.35	97.85 ± 34.81	0.894
Cr (mg/dL)	8.11 ± 2.42	7.86 ± 1.98	0.751	8.41 ± 3.01	8.04 ± 2.18	0.651
Ca (mg/dL)	9.58 ± 0.81	7.64 ± 1.09	**<0.001**	9.11 ± 1.49	8.12 ± 0.88	**<0.001**
P (mg/dL)	5.55 ± 1.14	4.34 ± 0.89	**<0.001**	5.74 ± 2.19	4.72 ± 0.91	**<0.001**
PTH (ng/L)	1443.87 ± 695.10	36.75 ± 18.74	**<0.001**	1671.48 ± 714.02	54.48 ± 20.63	**<0.001**
*Echocardiography findings*						
LVED diameter (cm)	4.09 ± 0.22	4.42 ± 0.23	0.715	4.26 ± 0.59	4.38 ± 0.70	0.781
LVES diameter (cm)	2.53 ± 0.57	2.35 ± 0.37	**0.042**	2.62 ± 0.47	2.42 ± 0.34	0.651
LVED septum thickness (cm)	1.09 ± 0.14	1.11 ± 0.15	0.812	1.11 ± 0.15	1.13 ± 0.13	0.907
LVED posterior wall thickness (cm)	1.05 ± 0.15	1.00 ± 0.15	0.924	1.04 ± 0.21	0.96 ± 0.36	0.759
EF (%)	59.25 ± 0.05	67.81 ± 4.04	**0.023**	58.37 ± 0.06	59.68 ± 20.46	0.312
Fractional shortening	40.58 ± 10.22	43.93 ± 6.85	0.192	41.09 ± 7.22	42.82 ± 6.54	0.642
End-diastolic volume	71.30 ± 11.33	69.13 ± 9.72	0.413	75.78 ± 6.39	73.71 ± 7.99	0.349
End-systolic volume	25.80 ± 5.96	21.56 ± 3.67	**0.008**	28.87 ± 5.69	24.76 ± 4.41	0.319
Ejection volume	45.50 ± 7.88	49.56 ± 7.80	**0.021**	49.91 ± 6.42	49.94 ± 7.51	0.891
IVRT	87.23 ± 12.50	79.87 ± 10.94	0.289	84.30 ± 8.26	78.90 ± 8.79	0.419
IVCT	79.81 ± 5.53	80.06 ± 8.84	0.569	77.30 ± 6.29	83.30 ± 4.86	0.241
ET	290.09 ± 23.13	313.05 ± 26.37	0.311	288.04 ± 22.69	303.11 + 26.37	0.572
MPI	0.45 ± 0.07	0.39 ± 0.06	**<0.001**	0.46 ± 0.04	0.40 ± 0.04	**0.011**

*Hgb* hemoglobin, *BUN* blood urea nitrojen, *Cr* kreatinin, *Ca* calcium, *P* phosphorus, *PTH* parathormone, *LVES* left ventricular end-systolic, *LVED* left ventricular end-diastolic, *EF* ejection fraction, *IVRT* isovolumetric relaxation time, *IVCT* isovolumetric contraction time, *ET* ejection time, *MPI* myocardial performance index

EF was 59.25 ± 0.05 preoperatively and 67.81 ± 4.04 postoperatively. Ejection volume was 45.50 ± 7.88 preoperatively and 49.56 ± 7.80 postoperatively. In Group 1, the postoperative value of EF and ejection volume increased significantly compared to the preoperative period (*p* = 0.023, *p* = 0.021, respectively) (Table 3).

The mean MPI decreased from 0.50 ± 0.07 preoperatively to 0.39 ± 0.04 postoperatively in group 1. The mean MPI decreased from 0.46 ± 0.06 preoperatively to 0.40 ± 0.04 postoperatively in group 2. In both groups, the postoperative value of MPI increased significantly compared to preoperatively (*p* < 0.001). There was no statistical difference between the preoperative and postoperative periods in other parameters in groups 1 and 2 (*p* > 0.05) (Table 3).

## Discussion

Studies have shown that parathyroidectomy performed in secondary hyperparathyroidism resistant to medical treatment due to chronic renal failure causes a significant reduction in cardiovascular diseases and mortality [17–19]. The current study analyzed the effect of total and subtotal parathyroidectomy for SHPT on cardiac functions through echocardiogram data. While echocardiogram findings showed improvement in both groups, the improvement was statistically significant in the total parathyroidectomy group, especially regarding myocardial performance index (MPI), EF, ejection volume, and end-diastolic volume.

The pathophysiology of the mechanism of formation of cardiovascular effects due to high parathyroid hormone and the resulting biochemical changes in the organism is not fully understood. However, there are various hypotheses regarding this. Due to high calcium and phosphorus levels, calcification of arterial vascular systems is found in approximately 80% of SHPT patients [20]. Although vascular calcification is closely related to cardiovascular diseases in the general population, considering that it occurs together with other cardiovascular risk factors, it is not clear to evaluate it as an independent predictive value [21]. On the other hand, another study showed that the mortality rate was higher in low iPTH and high serum calcium and phosphorus levels [22]. Furthermore, it has been reported that coronary arterial calcification decreases or does not progress after parathyroidectomy, and the risk of cardiovascular events decreases [23].

One of the most important reasons for the increased risk of cardiovascular disease and mortality in CKD patients is elevated fibroblast growth factor 23 (FGF23) and phosphate levels [24]. The increase in FGF23 levels is associated with cardiac fibrosis, left ventricular hypertrophy, and hypertension [25]. Therefore, treatment modalities for correcting FGF23 and phosphate levels are crucial in minimizing CKD-related cardiovascular risks.

Myocardial hypertrophy is seen in approximately 40% of early-stage CKD patients; this rate reaches up to 80% in end-stage CKD patients [26]. In vitro and in vivo studies demonstrated that FGF23 is directly related to cardiac remodeling [27, 28]. In an experiment with mice, intramyocardial or intravenous injection of FGF23 was shown to cause LVH through FGFR4-dependent activation of the calcineurin-nuclear factor of activated T-cells [29]. Thus, decreased FGF23 levels due to the normalized calcium and phosphorus balance after parathyroidectomy are associated with decreased risk of cardiovascular disease [30].

In the current study, when cardiac functions were evaluated after parathyroidectomy, we saw that only the end-systolic volume improved in favor of total parathyroidectomy between the two groups. In addition, when the two groups were evaluated regarding preoperative and postoperative cardiac functions, there was a significant improvement in LVES diameter, end-systolic volume EF, ejection volume, and MPI in the total parathyroidectomy group. In the subtotal parathyroidectomy group, only improvement in MPI was found. In a study designed similar to our study, a dramatic improvement was observed in echocardiogram data performed in the first year after parathyroidectomy, especially in left ventricular mass and left ventricular mass index, and in parameters, such as EF%, left ventricular end-diastolic diameter, left ventricular end-systolic dimension [31].

In this study, EF%, the leading indicator of systolic cardiac function, was evaluated. The total parathyroidectomy group observed an increase from 59.25 to 67.81%. In the subtotal parathyroidectomy group, improvement was observed from 58.37 to 59.68%. Another similar study showed that the %EF improved dramatically according to the echocardiogram results performed during the sixth postoperative month [32]. Furthermore, it has been proven that high PTH levels are associated with low EF%, decrease the afterload, and improve the EF% by normalizing PTH [33].

In the current study, MPI was also evaluated, unlike the classical parameters used to evaluate cardiac functions after parathyroidectomy. The MPI was defined by Tei in 1995 and is used to evaluate heart function, including systolic and diastolic time intervals. Since ejection time (ET), isovolumetric contraction time (ICT), and isovolumetric relaxation time (IRT) parameters are used in the formation of the MPI index; they provide sufficient information about myocardial functions. However, PMI is closely related to diastolic parameters and appears superior to traditional diastolic parameters in detecting impaired relaxation [34]. However, PMI varies inversely with EF; the higher the index value, the lower the EF, and vice versa [35]. In our study, MPI decreased from 0.45 ± 0.07 to 0.39 ± 0.04 in the group of patients who underwent total parathyroidectomy and from 0.46 ± 0.06 to 0.40 ± 0.04 in the group of patients who underwent subtotal parathyroidectomy, and it was statistically significant in both groups.

There are several limitations in the present study. Among these are the small number of patients included in the study and the single center’s retrospective planning of the study. Another limitation stems from the fact that the medical treatments used by the patients included in the study were due to secondary hyperparathyroidism and cardiovascular diseases, and the factors contributing to cardiovascular diseases needed to be fully addressed. Despite these, although other factors affecting cardiac functions were not mentioned in the study, it was shown that left ventricular functions and myocardial performance index improved in patients who underwent parathyroidectomy. However, we will see that the effect of parathyroidectomy on cardiac functions will be more clearly elucidated in the coming years with prospective multicenter and more homogeneous studies to be conducted. Besides all the limitations of the study, there are also advantages. The most important of these is that it is the only study in the literature in which the myocardial performance index, used to evaluate cardiac functions, is evaluated in patients who underwent parathyroidectomy due to secondary hyperparathyroidism. In this context, our study will contribute to the literature.

## Conclusion

In line with the information obtained from the echocardiogram data of the present study, it has been shown that there is an improvement in cardiac functions after parathyroidectomy. In the group that underwent total parathyroidectomy, improvement was observed in left ventricular end-systolic diameter, EF, ejection volume, and end-systolic volume. The statistical significance of these factors was not shown in subtotal parathyroidectomy. However, while the myocardial perfusion index, an important novel indicator of cardiac functions, demonstrated improvement in both groups, this improvement was more pronounced in patients who underwent total parathyroidectomy.
